# Educational background of nurses and their perceptions of the quality and safety of patient care

**DOI:** 10.4102/curationis.v38i1.1126

**Published:** 2015-04-30

**Authors:** Reece P. Swart, Ronel Pretorius, Hester Klopper

**Affiliations:** 1 School of Nursing Science, North-West University, South Africa

## Abstract

**Background:**

International health systems research confirms the critical role that nurses play in ensuring the delivery of high quality patient care and subsequent patient safety. It is therefore important that the education of nurses should prepare them for the provision of safe care of a high quality. The South African healthcare system is made up of public and private hospitals that employ various categories of nurses. The perceptions of the various categories of nurses with reference to quality of care and patient safety are unknown in South Africa (SA).

**Objective:**

To determine the relationship between the educational background of nurses and their perceptions of quality of care and patient safety in private surgical units in SA.

**Methods:**

A descriptive correlational design was used. A questionnaire was used for data collection, after which hierarchical linear modelling was utilised to determine the relationships amongst the variables.

**Results:**

Both the registered- and enrolled nurses seemed satisfied with the quality of care and patient safety in the units were they work. Enrolled nurses (ENs) indicated that current efforts to prevent errors are adequate, whilst the registered nurses (RNs) obtained high scores in reporting incidents in surgical wards.

**Conclusion:**

From the results it was evident that perceptions of RNs and ENs related to the quality of care and patient safety differed. There seemed to be a statistically-significant difference between RNs and ENs perceptions of the prevention of errors in the unit, losing patient information between shifts and patient incidents related to medication errors, pressure ulcers and falls with injury.

## Introduction

Quality patient care and safety is non-negotiable in healthcare (Chassin 1998; World Health Organization [WHO] 2011:29) and international studies recognise the link ‘between the educational background of nurses and the quality and safety of patient care’ (Aiken *et al.* 2003). The main aim of this study was to investigate the relationship between the educational background of nurses, as well as their perceptions of quality and safety in patient care in surgical units in private hospitals in Gauteng Province, South Africa (SA). This study formed part of an international collaborative research programme, Nurse Forecasting in Europe (RN4CAST), which aims to develop human resource forecasting models in nursing. As such, data on the workforce profile of nurses were collected across medical-, surgical-, midwifery and intensive care units, in both the public- and private health care sector in South Africa (SA). This article builds on and extends a body of research by the RN4CAST programme on the status of the practice environment of the nurse, levels of burnout, the quality and safety of patient care and the workload of nurses in medical- and intensive care units in SA (Blignaut 2012; Coetzee *et al.* 2013; Klopper *et al.* 2012; Pretorius & Klopper 2012). To achieve the aim of this study, the researchers investigated data collected in surgical units in the private healthcare sector in SA.

When referring to nurses in the context of this study, the researchers explored perceptions of both registered (RNs) and enrolled nurses (ENs) in terms of the quality and safety of care delivered. The reason for the focus on surgical wards in the private healthcare sector in Gauteng Province, was that although Gauteng is the smallest of the nine provinces in SA, it is home to almost 11.2 million people and is also the wealthiest and most populous per square metre (Statistics South Africa 2013). In addition, the most private healthcare beds and beneficiaries are located in this province (Matsebula & Willie 2007:163).

### Background and literature review

The South African healthcare system comprises a large public sector that caters for the needs of approximately 85% of the total population and a private sector that looks after the remaining 15% (Human Sciences Research Council 2008). Nursing numbers in the smaller private sector exceed those in the public sector and can probably be attributed to the healthier work environments in the private sector (Bateman 2010). Appropriate training of nurses is important in order to ensure high quality and safety of patient care (Aiken *et al.* 2003). In addition to that, Hoban (2003) emphasises that it is crucial to delegate patients according to the competency level of the nurses as this will ensure quality nursing care. Countries around the world are determined to improve the education of nurses as a means of improving the quality and safety of patient care (Van de Mortel & Bird 2010). Globally, nurses are motivated to improve their educational level to a baccalaureate level.

According to Pratt *et al*. (1993), inexperienced ENs exacerbate the workload of RNs. Studies conducted in other countries have revealed better patient outcomes where Baccalaureate-prepared RNs were responsible for most of the patient care. This can possibly be attributed to the fact that nurses who had been prepared at a baccalaureate level have stronger communication and problem solving skills (Johnson 1988) and ‘a higher proficiency in their ability to make nursing diagnoses and evaluate nursing interventions’ (Giger & Davidhizar 1990). Hospitals that had more technology available and adequate numbers of RNs on staff, showed increased performance on all processes related to patient care (Lucero, Lake & Aiken 2009).

Nurses are the backbone of the healthcare system and are critical to ensuring quality patient care and safety. Poor quality of care and safety are linked to the severe shortages of nursing human resources. The Health Systems Trust (2010) estimates a current shortage of 46.3% of nurses in the public health sector and 24% in the private health sector in hospitals in Gauteng (Hospital Association of South Africa [HASA] 2008). Although there has been an increase in the number of nurses (both RNs and ENs) trained in private hospitals in SA since 1998, regulatory constraints have had a negative impact and not nearly enough nurses are being trained (HASA 2010). Other problems concerning the quality of patient care in hospitals in SA include the misuse of services, errors that might have been avoided, a lack of or ineffective resources and records not being well kept (HASA 2010). In the private healthcare sector, Statistics South Africa (2007) reported that although only 7.8 million people have medical insurance, it seems that close to 15 million South Africans opted to use private healthcare services. In 2009, HASA stated that the sector will need 3756 more nurses to keep up with its current nursing ratios in the light of the increasing number of patients being seen.

It is further believed that the quality of care in hospitals and patient safety are deteriorating as a result of financial pressure, inadequate staffing and poor working conditions (Needleman *et al.* 2002). Adverse events such as falls, high mortality rates, injuries, hospital-acquired infections and pressure ulcers are also rising (Needleman *et al.* 2002). According to the World Health Organization (WHO 2012) one in every 10 patients admitted to a hospital in developing countries experiences an adverse event during their stay in hospital. Other factors related to poor quality of care in countries such as Canada, Germany, England, Scotland and the United States include: burnout of nurses, higher patient load (implying more acute patients per staff member); burdening nurses with non-nursing tasks; and poor management of hospitals (Aiken *et al.* 2001). Similar findings were also reported in studies conducted in SA (Coetzee *et al.* 2013; Klopper *et al.* 2012).

### Problem statement

Evidence suggests that there is a relationship between the quality of care and safety and the educational background of nurses (Aiken *et al.* 2003:1619; Giger & Davidhizar 1990; Johnson 1988). To that end, the severe shortage of nurses in SA and around the world further contributes to the endangerment of the quality and safety of care delivered to patients (Blignaut 2012:16). From the literature presented, it was evident that staff qualifications have a direct impact on the quality and safety of care delivered to patients. Nurses’ perceptions of the quality and safety of care delivered to patients can provide valuable information for patient outcomes and improvement of the overall standards of care. In order to increase the quality and safety of patient care in an ever-changing environment in SA, as well as to contribute to the growing body of literature on nurse forecasting in South Africa, an exploration of the relationship between the variables mentioned seems to be of vital importance. From the literature, the following research question arose: Is there a relationship between the educational background of RNs and ENs and their perceptions of the safety and quality of patient care in surgical units in private hospitals of the Gauteng Province?

#### Definition of key concepts

**Registered nurse:** According to s 30(1) of the South African Nursing Council's (SANC) *Nursing Act* No. 33 of 2005 (SANC; South Africa 2005), a ‘professional [*registered*] nurse is a person who is qualified and competent to independently practice comprehensive nursing in the manner and to the level prescribed and who is capable of assuming responsibility and accountability for such practice’.

**Enrolled nurse:** Section 30(2) of the *Nursing Act* (South Africa 2005) defines an enrolled [*staff*] nurse as ‘a person educated to practise basic nursing in the manner and to the level prescribed’.

**Educational background:** According to the South African Qualifications Authority (SAQA 2011), the qualification of the nurse, or educational background, is the formal recognition of the achievement of the required number and range of credits for a specific qualification. Two specific qualifications applied to this study, namely: the four-year degree or diploma in nursing that leads to registration as a registered nurse; and the two- year diploma leading to the registration as an enrolled nurse.

**Perceptions:** Blignaut (2012:22) describes perceptions as the basis of how a person sees and understands a concept and what is included in the mental image when referring cognitively to the same concept.

**Quality of care:** Pronovost *et al.* (2011:348) define quality measurement as the ‘lenses through which we quantitatively determine quality’. In this study, the nurses’ perceptions of the quality of care were measured using the 13 items of the RN4CAST questionnaire designed to explore the quality of patient care. Nurses were asked to indicate on a Likert scale the extent to which they agreed or disagreed with the statement (e.g. ‘In general, how would you describe the quality of care delivered to patients on your unit?’).

**Patient safety:** The WHO (2011) defines patient safety as ‘the prevention of errors and adverse events … associated with health care’. Furthermore, Scott (2003:13) states that the aim of patient safety implies flawless care or no mistakes. In this study, the nurses’ perceptions of the safety of care were measured using the 12 items of the RN4CAST questionnaire designed to explore the safety of patient care. Using a six-point Likert scale, nurses were asked to indicate the occurrence of incidents (‘never’ to ‘every day’) in patients in their unit (e.g. ‘patient received wrong medication, time or dose’).

## Research methods and design

### Research design

This study followed a descriptive correlation design. The independent variable, educational background and perceptions of the nurses in terms of the quality and safety of care delivered (dependent variables), were described and the differences in the variables between the groups were examined as they occurred in the natural setting (Burns & Grove 2009:239).

### Context

SA is divided geographically into nine provinces. The setting for this study was the private hospital sector in Gauteng Province; although Gauteng is the smallest of the nine provinces in SA, it is by far the wealthiest and most populous per square metre, with 12.78 million people in residence (Statistics South Africa 2013). For that reason, the most private healthcare beds, nursing workforce and medical aid beneficiaries are also located in this province. South African nurses are divided into three different categories, namely, RNs (also known as professional nurses); ENs (also known as staff nurses) and auxiliary nurses (NAs) (also known as nursing assistants). RNs have the option to enrol for a four-year degree at an accredited University, or a four-year diploma at an approved nursing college (SANC 2011a; South Africa 2005; Van Wyk 2006). The four-year programme enables graduates to register with the SANC as general, psychiatric and community nurses and midwives (Van Wyk 2006). ENs are trained at approved nursing colleges and students typically follow a two year in-service training programme. Both ENs and NAs practise under the direct or indirect supervision of a RN (South Africa 2005). According to the most recent statistics provided by SANC (2011b), there is approximately one RN for every 434 patients as opposed to one EN for every 995 patients in South Africa. In Gauteng Province, one registered nurse cares for 372 patients, whilst one enrolled nurse is available for every 861 patients. The statistics do not distinguish between the numbers for the public- and private healthcare sectors.

### Population and sample

Nurses working in surgical wards in private hospitals in Gauteng Province with a bed capacity > 100 were invited to participate in the study. Twenty-seven hospitals were included in the study and, because of the fact that nurses’ response rates to questionnaires are at best moderate, the researchers decided on an all-inclusive sample of RNs and ENs working in surgical units. From the staff list, 292 RNs and 306 ENs were invited to complete the questionnaire. A total of 304 fully-completed questionnaires were accepted for statistical evaluation, resulting in a 51% response rate. Of the 304 questionnaires received, the RNs completed 149 and the ENs 155. This resulted in a 51% response rate for both the RNs and the ENs.

### Data collection

#### Instrument

The questionnaire consisted of four sections with 118 questions across seven pages and typically took 15–20 minutes to complete. For the purposes of this study, only Sections B and D will be discussed. Section B was concerned with issues relating to the quality and safety of patient care in the unit, whilst demographic information of the nurses was collected in Section D. Twenty-five survey items measured nurses’ perceptions of the safety and quality of care in their units. A confirmatory factor analysis was conducted on the larger sample of data which included surgical units across SA (Klopper *et al.* 2012). Reliability was demonstrated by means of instrument homogeneity. Internal consistency, being a test of homogeneity, is determined by Cronbach's alpha (Chiang & Lin 2009:921). The Cronbach alphas for the subscales in this study (and when tested against the larger sample) ranged between 0.72–0.91 and were considered satisfactory. The reliability of the instrument was also demonstrated in several other studies (Coetzee *et al.* 2013; Klopper *et al.* 2012). To ensure validity of the study findings, current literature was evaluated, the questionnaire was scrutinised by content experts and the representativeness of the population in the RN4CAST study was considered to be of a high level.

Nurses were asked to identify, on a summated rating scale, the safety and quality of care that patients received in their unit. Practices related to patient safety that were explored consisted of: (1) staff mistakes that were held against them; (2) important patient information that was lost during shift changes; (3) ways to prevent recurrence of errors; (4) feedback based on incident reports; and (5) patient safety being considered a top priority by management. Nurses’ perceptions of incidents were also explored in terms of medication errors, occurrence of pressure ulcers, patient falls, hospital-acquired infections, patient complaints, verbal and physical abuse directed toward nurses and work-related injuries. The 19 items clustered into seven factors that included: (i) error prevention (4 items); (ii) losing patient information (2 items); (iii) staff mistakes (1 item); (iv) verbal abuse (3 items); (v) hospital-acquired infections (3 items); (vi) physical abuse (3 items); and (vii) patient incidents (3 items). The mean values for the factors and the reliability indices are reflected in Table 1.

**TABLE 1:** Mean scores and reliability indices of the factors identified (*N* = 304).

Factor	Number of items	Total sample mean (SD)	Cronbach alpha
Error prevention (*n* = 300)	4	3.89 (0.78)	0.74
Losing patient information (*n* = 299)	2	2.73 (1.28)	0.83
Staff mistakes (*n* = 296)	1	3.25 (1.27)	-
Verbal abuse (*n* = 296)	3	2.12 (1.57)	0.83
Hospital-acquired infections (*n* = 292)	3	0.96 (1.23)	0.91
Physical abuse (*n* = 296)	3	0.77 (1.11)	0.78
Patient incidents (*n* = 297)	3	0.98 (1.03)	0.72

#### Procedure

Data collection took place on-site over a period of three months (May–July 2009) and the process was coordinated by one of the researchers. Appointments were scheduled with the nursing service manager of each of the 27 hospitals that were included in the study. During the meeting, the researcher explained the process and the questionnaire, after which a fieldworker was appointed and trained in order to assist with both the distribution and collection of the questionnaires in each of the participating surgical units.

### Data analysis

Descriptive data were analysed using SPSS 16.0 (SPSS Inc., Chicago IL 2007) and included mean scores and reliability indices as well as frequencies and percentages for questions related to the quality and safety of patient care. A Pearson chi-square test was also conducted to examine the relationship between the educational background of nurses and their perceptions of the quality and safety of patient care. Associations amongst the study variables were estimated using hierarchical linear modelling (HLM) in Statistical Analysis System (SAS). According to McCoach (2010), a large proportion of the data in the social sciences are hierarchical in nature and when people are clustered within naturally-occurring units, their responses are likely to exhibit some degree of relatedness. Because the data in this study were hierarchical in nature, with nurses working in surgical units within private hospitals, HLM was performed.

## Results

The demographic profile of the participants is illustrated in Table 2. From the results, it was evident that the majority of the participants in both the RN (*n* = 142; 96.6%) and EN (*n* = 141; 93.4%) categories were women. When asked about their age, the RNs’ ages ranged from 24 to 63 years (mean = 43.01, SD = 9.53); and the ENs’ ages ranged from 22 to 54 years (mean = 40.75, SD = 13.74). Nineteen (13.4%) of the RNs were trained at a baccalaureate level and 123 (86.6%) at a diploma level. Most of the nurses in both categories were also employed as fulltime workers in the unit (RNs: *n* = 137, 93.8%; ENs: *n* = 140, 95.2%).

**TABLE 2:** Demographic characteristics of the participants (*N =* 304).

Variables	Total sample (%)	
	RN (*n* = 149)	EN (*n* = 155)
Age (mean ± SD)	43.01 (9.53)	40.75 (13.74)
Female	142 (96.6%)	141 (93.4%)
Male	5 (3.4%)	10 (6.6%)
Degree	19 (13.4%)	-
Diploma	123 (86.6%)	-
Full-time employees	137 (93.8%)	140 (95.2%)
Part-time employees	9 (6.2%)	7 (4.8%)

RN, registered nurses; EN, enrolled nurses.

When asked about their general opinion on the quality of care delivered to patients on their ward, 61 (50.8%) of the RNs rated service as good and 91 (60.3%) of the ENs rated service as excellent (refer to Table 3). Chi-square analysis revealed a significant difference between RNs’ and ENs’ general opinion of the quality of care delivered: *X*^2^(3, *N* = 271) = 23.1, *p* = < 0.001.

**TABLE 3:** In general, how would you describe the quality of nursing care delivered to patients on your ward?

Variables	Registered nurses (*n* = 120)		Enrolled nurses (*n* = 151)	
	Number	%	Number	%
Poor	1	0.8	1	0.7
Fair	18	15	6	4.0
Good	61	50.8	53	35.1
Excellent	40	33.3	91	60.3

Moreover, 51% (*n* = 77) of the ENs rated the overall grade on patient safety in their ward as very good, whilst 75 (51%) of the RNs rated it as acceptable (refer to Table 4). As before, the chi-square test revealed a significant difference between RNs’ and ENs’ overall grade of safety in their wards: *X^2^* (3, *N* = 298) = 34.1, *p* = < 0.001.

**TABLE 4:** Please give your unit an overall grade on patient safety.

Variables	Registered nurses (*n* = 147)		Enrolled nurses (*n* = 151)	
	Number	%	Number	%
Failing	6	4.1	2	1.3
Poor	37	25.2	17	11.3
Acceptable	75	51	55	36.4
Very good	29	19.7	77	51

Deterioration of the quality of care was less commonly reported by the ENs, with 108 (74.5%) of the ENs agreeing that the quality of care in their hospitals had improved in the past year. For the RNs, an equal percentage of nurses (37.5%; *n* = 54) were of the opinion that the quality of care had either remained the same or had improved in the past year (refer to Table 5). The chi-square analysis revealed a significant difference between RNs’ and ENs’ perceptions about the quality of care in their hospitals: *X^2^* (2, *N* = 289) = 42.4, *p* = < 0.001.

**TABLE 5:** In the past year would you say the quality of care in your hospital has …?

Variables	Registered nurses (*n* = 144)		Enrolled nurses (*n* = 145)	
	Number	%	Number	%
Deteriorated	36	25	9	6.2
Remained the same	54	37.5	28	19.3
Improved	54	37.5	108	74.5

Close to 44% (*n* = 65) of the ENs were very confident that their patients were prepared adequately to manage their care at home upon discharge, whilst 81 (55.1%) of the RNs were confident (refer to Table 6). Yet again, the chi-square test revealed a significant difference between RNs’ and ENs’ confidence in patients managing their care when they are discharged from hospital: *X^2^* (3, *N* = 295) = 12.0, *p* = < 0.001.

**TABLE 6:** How confident are you that your patients are able to manage their care when discharged?

Variables	Registered nurses (*n* = 147)		Enrolled nurses (*n* = 148)	
	Number	%	Number	%
Not at all confident	3	2	5	3.4
Somewhat confident	25	17	16	10.8
Confident	81	55.1	62	41.9
Very confident	38	25.9	65	43.9

The analysis of the relationship between the variables yielded the following results. As presented in Table 7, HLM indicated a statistically-significant difference (level of significance at 5%) between RNs and ENs in that enrolled nurses seemed to agree with the actions taken to prevent errors from happening in surgical units (*p* < 0.001). Registered nurses (RNs) neither agreed nor disagreed with the statements. Looking at the sub-scale, losing patient information, there was a statistically-significant difference between RNs en ENs responses (*p* = 0.05). ENs seemed to disagree more with statements that important information is lost during shift changes or when transferring patients from one unit to the next. The mean score for the RNs, at 2.91, leans toward neither agreeing nor disagreeing with the statements on losing patient information. In terms of staff feeling that their mistakes are held against them, the RNs’ mean score of 3.08 indicated that they neither agreed nor disagreed with the statement. The ENs, however, with a mean score of 3.47, seemed to lean toward agreeing with the statement that their mistakes are held against them. In terms of verbal abuse, both RNs and ENs agreed that verbal abuse occurred once a month or less, with the RNs (mean = 2.25) having a slighter higher mean score than the ENs (mean = 2.08). Looking at the mean scores for physical abuse, both RNs (mean = 0.63) and ENs (mean = 0.89) seem to agree that incidents occur only a few times or less per year. Finally, when reporting on patient incidents in terms of medication errors, pressure ulcers and falls with injury, a statistical significance (*p* < 0.001) was found between RNs and ENs. RNs (mean = 1.08) indicated an occurrence of a few times per year or less, whilst the ENs, with a mean score of 0.80, were of the opinion that these incidents never occurred in their units.

**TABLE 7:** Results of Hierarchical Linear Models for educational background and the safety and quality of patient care.

Variables	Registered nurses		Enrolled nurses		*t*-value	*P*-value
	Mean	Standard error	Mean	Standard error		
Error prevention	3.69	0.07	4.05	0.05	−4.89	< 0.001
Losing patient information	2.91	0.12	2.47	0.19	2.23	0.05
Staff mistakes	3.08	0.09	3.47	0.18	−2.03	0.07
Verbal abuse	2.25	0.12	2.08	0.31	0.51	0.62
Hospital-acquired infections	0.79	0.11	1.06	0.14	−1.43	0.19
Physical abuse	0.63	0.12	0.89	0.14	−1.33	0.22
Patient incidents	1.08	0.09	0.80	0.10	5.03	< 0.001

## Ethical considerations

A formal, written proposal of the project that complies with the Declaration of Helsinki, was approved by the Health Research Ethics Committee of the North-West University under number: NWU-0015-08-S1. Approval was also obtained from the Ethics Committees of the private hospital groups that participated in the study.

### Potential benefits and hazards

The benefits of the study include a better understanding of the relationship between nurses’ educational background in the private hospital context in SA and their perceptions on the safety and quality of patient care. There was no risk or discomfort to any of the participants in this study.

### Recruitment procedures

Participation in this study was voluntary and refusal to participate was without penalty or loss of benefit to which any participant may otherwise have been entitled. Participants could also withdraw from the study at any time without penalty.

### Informed consent

Participants were informed of the aim of the study and consent was obtained in recognition of voluntary participation and the right to protection. The informed consent process was coordinated by the researchers and a trained fieldworker assisted with the distribution and collection of the questionnaires and informed consent documents at each site.

### Data protection

The researchers have access to the raw data and all the data will be kept for a period of five years in a lockable cupboard to prevent unauthorised entry. Data used on the electronic spread sheets for the purpose of analysis is stored in files that have access codes.

## Trustworthiness

### Reliability

Reliability was demonstrated by means of instrument homogeneity. The Cronbach alphas for the subscales in the study ranged between 0.72–0.91 and was considered satisfactory.

### Validity

Validity of the findings was ensured by reviewing current literature, scrutiny of the questionnaire by content experts and representativeness of the population under study.

## Discussion

This study compared the perceptions of RNs and ENs on the safety and quality of patient care in the surgical units of private hospitals in Gauteng Province. Perceptions shape the foundation for all human action and nurses’ perceptions lead to actions that affect patient safety and the quality of care, all of which are critical to the provision of healthcare (Mwachofi, Walston & Al-Omar 2011:274).

### Outline of the results

Findings from this study indicated that SA, as is the case with the rest of the world, has a substantial nursing workforce aged 40 years and older that is predominantly female (Graham & Duffield 2010:44). The positive ratings of the patients’ preparedness for discharge amongst the RNs and ENs correlate with the findings of a similar study conducted by Aiken *et al.* (2001:49) in five countries and could possibly be attributed to longer hospital stays. From the results, it was also evident that RNs were more likely to report on incidents such as medication errors, occurrence of pressure ulcers and hospital-acquired infections. ‘Medication errors have serious direct and indirect results, and are usually the consequence of breakdowns in a system of care’ (Mayo & Duncan 2004:209). Several studies, however, demonstrate underreporting of medication errors amongst nurses (Gladstone 1995:628; Wakefield *et al.* 2001:128). Wakefield *et al.* (2001:129) cite several reasons for underreporting. These include the following: organisations relying on self-reporting of errors such as incident reports can miss up to 95% of medication errors; nurses’ inability to recognise errors; fear of punishment; disagreement over the definition of an error; and time to complete reports. The general positive rating of medication error occurring a few times a year or less in this study can probably be accredited to the fact that nurses were unable to detect errors, had the perception that certain errors will not harm patients, or had a fear of the consequences associated with reporting of errors (Wakefield *et al.* 2001:130). According to Mwachofi *et al.* (2011:276), organisational processes such as error-reporting are considered to be critical with regard to the influence they exert on nursing perceptions. Moreover, nurses’ willingness to report errors varies by severity, although most nurses are willing to report errors on all levels. A study conducted in critical care units in private, public and university hospitals in Turkey, found that private hospitals had more quality management and patient safety programmes and were also less likely to have punitive responses to errors reported, which could contribute to the positive ratings from ENs on error prevention in this study (Herdman & Badir 2008).

Contrary to the findings of several international studies (O’ Connell, Macdonald & Kelly 2008; O’ Connell & Penney 2001), ENs seemed to agree that patient information is not lost during shift changes or when transferring patients between wards. RNs, on the other hand, seemed indifferent. According to Chaboyer *et al.* (2009:136), accurate communication during handover is a critical element in the quality and safety of care that patients receive. Most international literature cites the quality of information during handovers as being poor, inaccurate or incomplete (O’ Connell *et al.* 2008:8). Such findings highlight the deficit and inefficiencies that have been associated with loss of information during nurse handovers. This study did not ask nurses about the quality of the information during nurse handovers; however, it may be useful to explore nurses’ perceptions regarding this.

RNs indicated that verbal abuse toward nurses by patients and/or families occurred once a month or less. According to Aiken *et al.* (2001:50), the current climate of care in hospitals is a major contributor to unsatisfied patients which, in turn, results in frustration that is likely to compromise the civility of the work environment. When reporting on physical abuse toward nurses by patients and/or family and staff, both the RNs and ENs indicated that such incidents occurred only a few times a year or less. In a study conducted by Day, Minichiello and Madison (2007:279), the authors found that almost three-quarters of the study population (*n* = 262) reported that they had been subjected to some form of physical abuse from their patients. The issue of workplace violence has received attention worldwide in the last decade. The low report of physical abuse from nurses in this study, when compared to international literature, can probably be attributed to nurses perceiving high levels of abuse from patients as ‘part of the job’. Day *et al.* (2007:280) questioned whether unmet patient needs and high expectations are not the cause of generating high levels of hostility and disrespect toward the most visible and available health practitioner, namely, the nurse. In terms of the objective of this study there seemed to be a statistically-significant difference between perceptions of RNs and ENs regarding the prevention of errors in the unit, loss of patient information between shifts and patient incidents related to medication errors, pressure ulcers and falls with injury.

## Limitations of the study

This article is based on a secondary analysis of the data. The study was conducted in the private hospital sector in Gauteng Province, limiting the findings to that context. Another limitation is that in a self-administered questionnaire, the provision of additional information (such as the quality of information during handovers) limits the results to the items asked. It is regrettable that the quality of the information during shift changes or transfers to other units was not explored.

## Conclusion

Nurses play a major role as primary patient contact and have a vital role in ensuring the quality and safety of patient care. The results of this study suggest that nurses (both registered and enrolled) have favourable perceptions of the quality and safety of patient care delivered in surgical units in private hospitals in Gauteng. It is strongly suggested that several of the findings that can affect the quality and safety of patient care, such as recording of medication errors, be addressed by management. To that end, the findings provide a glimpse of one of the critical professions’ perceptions on the safety and quality of care in surgical units in private hospitals.
